# Engineering circular bacteriocins: structural and functional effects of α-helix exchanges and disulfide introductions in circularin A

**DOI:** 10.3389/fmicb.2024.1337647

**Published:** 2024-02-15

**Authors:** Fangfang Liu, Auke J. van Heel, Oscar P. Kuipers

**Affiliations:** ^1^Department of Molecular Genetics, Groningen Biomolecular Sciences and Biotechnology Institute, University of Groningen, Groningen, Netherlands; ^2^Omnicin Therapeutics, Groningen, Netherlands

**Keywords:** circular bacteriocins, circularin A, bacteriocin engineering, peptide scaffold, α-helix replacement, disulfide bond, biosynthesis, bioactivity

## Abstract

Circular bacteriocins form a distinct group of antimicrobial peptides (AMPs) characterized by their unique head-to-tail ligated circular structure and functional properties. They belong to the ribosomally synthesized and post-translationally modified peptide (RiPP) family. The ribosomal origin of these peptides facilitates rapid diversification through mutations in the precursor genes combined with specific modification enzymes. In this study, we primarily explored the bacteriocin engineering potential of circularin A, a circular bacteriocin produced by *Clostridium beijerinckii* ATCC 25752. Specifically, we employed strategies involving α-helix replacements and disulfide bond introductions to investigate their effects on both biosynthesis and bioactivity of the bacteriocin. The results show the feasibility of peptide engineering to introduce certain structural properties into circularin A through carefully designed approaches. The introduction of cysteines for potential disulfide bonds resulted in a substantial reduction in bacteriocin biosynthesis and/or bioactivity, indicating the importance of maintaining dynamic flexibility of α-helices in circularin A, while reduction of the potential disulfide in one case increased the activity. The 5 α-helices of circularin A were respectively replaced by corresponding helices from another circular peptide, enterocin AS-48, and modestly active peptides were obtained in a few cases. Overall, this study provides valuable insights into the engineering potential of circular bacteriocins as antimicrobial agents, including their structural and functional restrictions and their suitability as peptide engineering scaffolds. This helps to pave the way for the development of novel antimicrobial peptides with tailored properties based on circular bacteriocins.

## Introduction

Bacteriocins are ribosomally synthesized antimicrobial peptides, proteins or proteinaceous complexes produced by a vast majority of bacterial species (Héchard and Sahl, 2002; Kawai et al., 2004; Rebuffat, 2012; Arnison et al., 2013; Darbandi et al., 2022). These antimicrobial products provide a defense mechanism for the producer bacteria to compete for space and resources in their surroundings (Cesa-Luna et al., 2020; Darbandi et al., 2022). Regarding the biosynthesis of bacteriocins, especially within the family of ribosomally synthesized and post-translationally modified peptides (RiPPs), these peptides are typically first synthesized as inactive precursors, which then undergo post-translational modifications (PTMs) to acquire their biological activity. RiPPs bacteriocins exhibit a broad range of structural diversity, which can be attributed to the wide variety of post-translational modifications (PTMs) they undergo. As natural products involved in species competition, bacteriocins also display varying antimicrobial specificity, including both narrow-spectrum bacteriocins that target specific bacteria (van Belkum et al., 2011) and broad-spectrum bacteriocins that can target a wide range of bacteria (Jack et al., 1995; Simons et al., 2020; Darbandi et al., 2022). This diversity makes bacteriocins desirable for a variety of applications. In particular, bacteriocins are of great interest for their potential use in food preservation by inhibiting the growth of foodborne pathogenic and spoilage bacteria (Verma et al., 2014; Ng et al., 2020), such as *Listeria monocytogenes, Bacillus cereus, Clostridium perfringens* and *Staphylococccus aureus* (Bintsis, 2017). Additionally, there is growing interest in exploring bacteriocins as an alternative to traditional antibiotics, considering the urgent need to combat the rapid development of antibiotic resistance (Cotter et al., 2013; Kayalvizhi, 2016; Benítez-Chao et al., 2021). As an increasing number of bacteriocin PTM enzymes have been characterized, bacteriocin engineering has demonstrated great potential for incorporating certain structural features into target peptides to generate new-to-nature compounds with desired properties.

**Figure 1:**
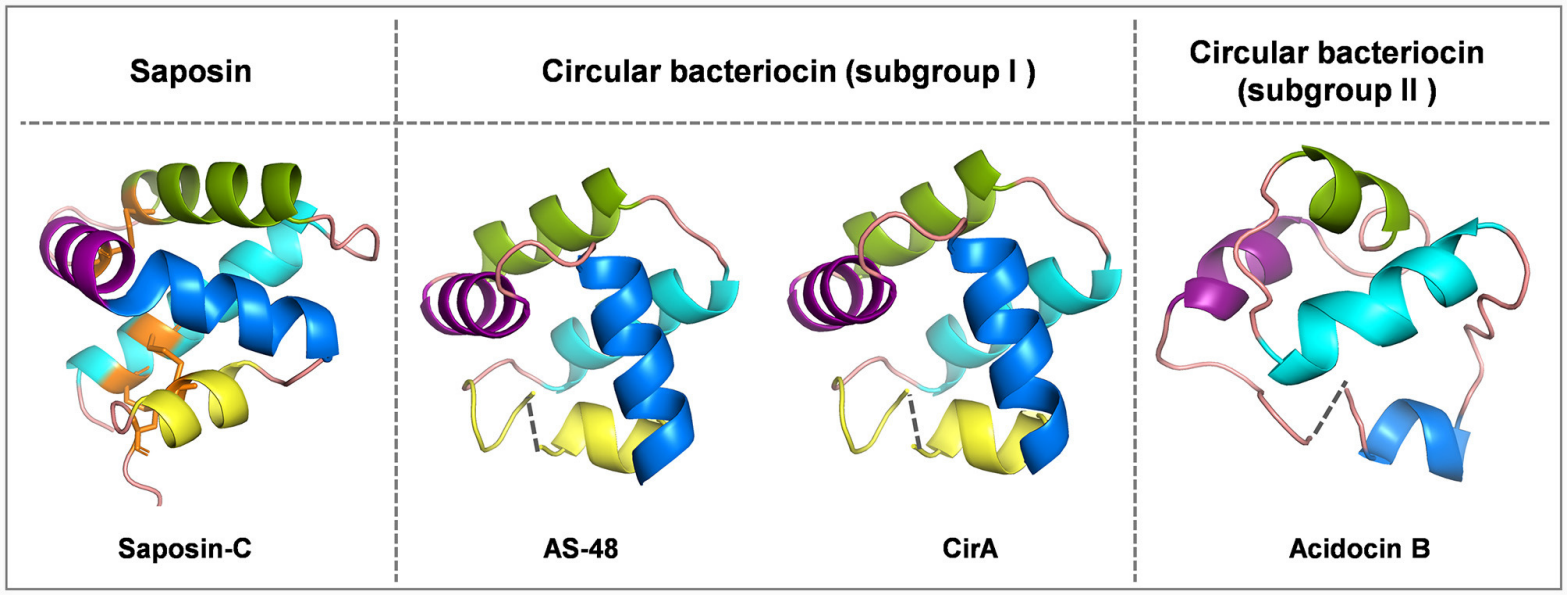
Cartoon representations of saposin-C, subgroup I circular bacteriocins AS-48 and circularin A, and subgroup II circular bacteriocin acidocin B created with PyMOL. Subgroup I circular bacteriocins generally adopt a saponin-like fold, whereas subgroup II circular bacteriocins have a closely related α-helical bundle structure. To depict directionality, each helix is colored differently starting from the N-terminus, cyan: α1-helix, green: α2-helix, purple: α3-helix, blue: α4-helix and yellow: α5-helix. The dashed lines indicate the ligation sites in circular bacteriocins. The peptide structure of saposin-C was downloaded from UniProt. There is no circularization in saposin-C, but three disulfide bridges stabilize its structure, and these three disulfide bridges are indicated in orange: C5-C78 (loop and α1 linkage), C8-C72 (α1 and α5 linkage) and C36-C47 (α2 and α3 linkage). The peptide structures of circular bacteriocins (including AS-48, circularin A and acidocin B) were modeled and downloaded from the SWISS-MODEL website.

Circular bacteriocins form a unique group of bacteriocins that are characterized by head-to-tail ligation, which is formed by a covalent bond connecting the N- and C-termini of the peptide. The circular topology confers circular bacteriocins great stability across a wide range of pH and temperature conditions, and enhances their resistance to proteolytic degradation (Perez et al., 2018). There are around 20 circular bacteriocins that have been characterized. Based on their biochemical properties such as peptide hydrophobicity, net charge and isoelectric point (pI), circular bacteriocins can be divided into two major subgroups: subgroup I circular bacteriocins are highly cationic and have high pI values, while subgroup II circular bacteriocins have comparatively lower pI values and display high hydrophobicity (Perez et al., 2018). To date, five circular bacteriocins have been structurally elucidated, including enterocin AS-48 (Sánchez-Barrena et al., 2003), carnocyclin A (Martin-Visscher et al., 2009) and enterocin NKR-5-3B (Himeno et al., 2015) (subgroup I), as well as acidocin B (Acedo et al., 2015) and plantacyclin B21AG (Gor et al., 2020) (subgroup II). Despite limited sequence similarity, most circular bacteriocins share a common structural fold known as the saposin-like fold consisting of four or five α-helices, and the circularization site is typically found within an α-helical structure (Martin-Visscher et al., 2009) (Figure 1).

Saposins are a group of four proteins (i.e., saposins A–D) derived from a single, larger precursor protein prosaposin produced in humans that are involved in sphingolipid catabolism within the lysosome (Vaccaro et al., 1999). They share a structural motif called the saposin fold, consisting of four or five α-helices and three disulfide bridges that stabilize their structure (Bruhn, 2005). Interestingly, this structural motif characteristic of saposins is found in a larger protein superfamily called saposin-like peptides (SAPLIPs), which is a diverse family of lipid-interacting proteins (Bruhn, 2005). Unlike the saposin fold of SAPLIPs, there are no disulfide bonds in bacteriocins to stabilize their saposin-like folds (Towle and Vederas, 2017). Disulfide bonds not only contribute to the structural stability of saposins and SAPLIPs, but also appear to be critical for their biological activity (Vaccaro et al., 1999). It has been shown that reduction of the disulfide bonds can result in a significant reduction in the ability of saponins to stimulate sphingolipid hydrolysis (O'Brien and Kishimoto, 1991; Suzuki, 1995), as well as a substantial decrease in the antimicrobial and cytolytic activities of NK-lysin (Andersson et al., 1996), a representative member of SAPLIPs. In contrast, circular bacteriocins appear to be stabilized by hydrophobic side-chain interactions upon peptide circularization, which helps maintain their helical structure and saposin-like fold.

The unique properties of circular bacteriocins, such as their exceptional structural stability and broad-spectrum antimicrobial activity (Gabrielsen et al., 2014), have attracted great scientific interest in elucidating their biosynthetic mechanisms, particularly their circularization. The understanding of biosynthetic mechanisms could allow the exploration of the great potential of circular bacteriocins in biotechnological applications, specifically the rational design of novel peptides with desired properties through peptide engineering. Although peptide engineering of circular bacteriocins may yield peptides with enhanced stability and target specificity, this requires a comprehensive understanding of bacteriocin structure-function relationships. However, understanding these relationships remains a great challenge in ongoing bacteriocin research (Snyder and Worobo, 2014; Gor et al., 2020).

Circularin A, a circular bacteriocin belonging to the subgroup I circular bacteriocins, is produced by *Clostridium beijerinckii* ATCC 25752. It is composed of a 3-amino acid (aa) leader sequence and a 69-aa core peptide (Table 1), and has been shown to possess potent antimicrobial activity against a wide range of Gram-positive bacteria, with a notable effectiveness against Clostridia (Kemperman et al., 2003). Previously, we successfully developed an efficient system for mutagenesis studies of circularin A in *Lactococcus lactis* NZ9000 by co-expressing its structural gene (pNZ-*cirA*) and biosynthetic cluster (pTLR4-*cirBCDE*) using the convenient nisin-controlled expression (NICE) system (Liu et al., 2022). With this circularin A production platform and the methodology developed previously (Liu et al., 2022), we here explore the possibility of circular bacteriocin engineering by examining the promiscuity of circularin A biosynthetic proteins to create hybrid peptides containing large exogenous peptide fragments. These fragments could potentially impart desired properties (e.g., altered target specificity) to circular bacteriocins. Moreover, we also investigated the effect of introducing potential disulfide bonds (causing structural constraints) in circularin A on bacteriocin biosynthesis and bioactivity. To assess the successful production and circularization, we relied on the combined approach of the activity assay and SDS-PAGE gel analysis. As reported previously (Liu et al., 2023), the activity assay is the most sensitive method for testing successful circularization of circularin A variants, since unmodified linear peptides are readily degraded by intracellular peptidases in *L. lactis*, leading to either full degradation or partial degradation (yielding a 25-aa C-terminal peptide fragment of circularin A variants) of the variant peptides, which could be shown with SDS-PAGE. Moreover, results obtained from SDS-PAGE also serve as supplementary evidence for variants that are still produced but have lost activity. The combination of the activity assay and SDS-PAGE ensures a robust investigation. To our knowledge, this is an unprecedented attempt at peptide engineering of circular bacteriocins using this approach. Our results provide valuable insights into the peptide engineering potential of circular bacteriocins and promote further exploration of circular bacteriocin biosynthesis in this context.

**Table 1:** Mutational analysis of α-helix replacement between circularin A and enterocin AS-48.

**Peptide variant**	Amino acid sequence^*^
Wild-type CirA	MFL*-VAGAL*GVQ*TAAATTIVNVIL*NAGT*LVTVLGIIASIASGGAGTLMT*IGWAT*FKATVQKLAKQ*S*MARAIAY*
α1-helix replacement	MFL-VAGALGVQAAVAGTVLNVVENAGTLVTVLGIIASIASGGAGTLMTIGWATFKATVQKLAKQSMARAIAY
α2-helix replacement	MFL-VAGALGVQTAAATTIVNVILNAGTVTTIVSILTAVASGGAGTLMTIGWATFKATVQKLAKQSMARAIAY
α3-helix replacement	MFL-VAGALGVQTAAATTIVNVILNAGTLVTVLGIIASIASGGLSLLAAIGWATFKATVQKLAKQSMARAIAY
α4-helix replacement	MFL-VAGALGVQTAAATTIVNVILNAGTLVTVLGIIASIASGGAGTLMTIGWATIKAYLKKEIKKKSMARAIAY
α5-helix replacement	MFL-MAKEFGVQTAAATTIVNVILNAGTLVTVLGIIASIASGGAGTLMTIGWATFKATVQKLAKQSKRA***VIA*****W**

^*^The presented amino acid sequences are supposed to be the precursor sequences, while the sequence of MFL represents the leader sequence found in the wild-type circularin A. The predicted sequences of 5 α-helices in the wild-type circularin A are indicated in italics and underlined. The portions of AS-48 that were introduced by replacing their circularin A counterparts are highlighted in bold and underlined, and the rest of the peptide sequence is from circularin A. It is important to note that due to the circular nature of the peptide, the first and last parts of the peptide form a single α-helix.

## Materials and methods

### Bacterial strains and growth conditions

*Lactococcus lactis* NZ9000 was used as the host strain for molecular cloning and bacteriocin expression in this study. It was cultivated at 30°C in GM17 broth, which consisted of M17 broth supplemented with 0.5% (w/v) glucose. To select positive transformants on solid medium, GM17 broth was supplemented with 1.5% (w/v) agar and appropriate antibiotics. When needed, chloramphenicol and erythromycin were used at 5 μg/ml each for *L. lactis* NZ9000.

The indicator strains used in this study include *Lactobacillus sake* ATCC 15521, *Enterococcus faecalis, Bacillus subtilis, Listeria monocytogenes* and *Lactococcus lactis* IL1403. Specifically, *Lb sake* ATCC 15521 was grown at 30°C in De Man Rogosa and Sharpe broth (MRS); *E. faecalis* was grown at 37°C in GM17 broth; *B. subtilis* was grown at 37°C in Luria–Bertani (LB) broth with vigorous agitation (220 rpm); *Listeria monocytogenes* was grown at 30°C in the Brain Heart Infusion (BHI) broth; *L. lactis* IL1403 was grown at 30°C in GM17 broth. For bacterial growth of indicator strains on plates in activity assays, 0.8% (w/v) agar was added to the medium. All the media and chemicals were purchased from Sigma-Aldrich unless otherwise specified.

### Molecular cloning and site-directed mutagenesis in *cirA*

The techniques of standard molecular cloning were performed as previously reported (Green et al., 2012). Site-directed mutagenesis was designed and performed to introduce the desired mutations in *cirA* based on a previously reported two-plasmid production system (pNZ-*cirA*+pTLR4-*cirBCDE*) (Liu et al., 2022). All designed primers (Supplementary Table S1) were purchased from Biolegio (Nijmegen, the Netherlands). PCR products were isolated using the NucleoSpin gel & PCR cleanup kit (Bioke, Leiden, the Netherlands) and ligation of DNA fragments was performed using either Gibson Assembly Master Mix or USER Enzyme (Bioke, Leiden, the Netherlands) following the manufacturers' instructions. Subsequently, the ligation mixture was desalted and subsequently transformed into “empty” *L. lactis* NZ9000 cells via electroporation using a Gene-Pulser (Bio-Rad), as previously described (Holo and Nes, 1989). Agar plates supplemented with 5 μg/mL chloramphenicol were used to select for plasmid presence. A few colonies resulting from this transformation were selected and inoculated into liquid GM17 medium supplemented with 5 μg/ml chloramphenicol, then cultivated overnight at 30°C. The overnight culture was subjected to plasmid isolation using the NucleoSpin Plasmid EasyPure kit. Subsequently, successful integration of the desired mutation was confirmed by gene sequencing (Macrogen Europe). Finally, the correctly sequenced *cirA* mutant (pNZ-*cirA*^mut^) was transformed into *L. lactis* NZ9000 cells, which already harbored genes encoding circularin A biosynthetic proteins (pTLR4-*cirBCDE*). The constructed strains were stored as 20% (v/v) glycerol stocks at -80°C.

### Bacteriocin production and C18 open-column purification

For bacteriocin production, slight modifications were made to the expression conditions of *cirA* mutants compared to the previously reported method (Liu et al., 2022), and two rounds of nisin induction were employed during peptide expression (Liu et al., 2023). Briefly, the expression host (*L. lactis*) was first grown overnight in the GM17 medium at 30°C. The following day, cells from the 5-mL fresh overnight culture were spun down and transferred into 100-mL minimal medium supplemented with 2.2% glucose and 5 ng/mL nisin for the initial induction. After being grown at 30°C for approximately 4 h (OD600: 0.3–0.5), an additional induction of 5 ng/mL nisin was introduced to the cell culture. The cells were then incubated at 30°C for 16–20 h. Finally, the cell culture was subjected to centrifugation at 8,000 g for 15 min, and the resulting supernatant was collected for subsequent C18 open-column purification. The C18 purification procedure was conducted according to the previously reported method (Liu et al., 2022). This method involved eluting the peptide in four elution gradients, including fractions eluted with 20%, 50%, 80%, and 100% solvent. For each elution fraction obtained from C18 purification, the peptide samples were first freeze-dried and then dissolved in a small volume of a 50% acetonitrile solution to concentrate 1,000-fold (e.g., a volume of 30 μL was used for peptide purified from 30 mL of cell culture). The concentrated samples were stored at 4°C for further analysis such as mass spectrometry and protein gel analysis.

### MALDI-TOF and tricine-SDS-PAGE

After purification, MALDI-TOF (matrix-assisted laser desorption/ionization-time of flight) and tricine-SDS-PAGE (tricine-sodium dodecyl sulfate–polyacrylamide gel electrophoresis) were employed to evaluate the production of peptide variants as previously reported (Liu et al., 2022). For MALDI-TOF analysis, 1-μl concentrated peptide sample was applied, using the same method as previously described (Liu et al., 2023). The amount of peptide used for tricine-SDS-PAGE analysis was purified from 20 mL of cell culture, i.e., 20-μl concentrated peptide sample. Specifically, the gels, consisting of a 16% separating gel and a 4% stacking gel, were prepared as previously reported (Schägger, 2006). For sample preparation, 5-μL loading buffer (10% SDS, 0.5% Bromophenol blue, 50% glycerol, 250 mM Tris-HCl, pH6.8) was mixed with the peptide sample, and the resulting mixture was heated at 50°C for 30 min. Additionally, a 5-μL portion of Unstained Low Range Protein Ladder (PageRuler, Thermo Fisher) was included as a protein marker and run alongside the peptide samples. After gel separation, the staining and destaining procedures were conducted following the previously reported method (Liu et al., 2022).

### Antimicrobial activity tests

The antimicrobial activity of engineered peptide variants was evaluated using the colony overlay assay, which involved two layers: the first layer (bottom layer) facilitated the bacterial growth and peptide expression of the bacteriocin-producing strain, while the second layer (top layer) was seeded with the indicator strain, as reported previously (Liu et al., 2022). Typically, for the first layer, the growth medium was GSM17 medium containing 0.5% glucose, 0.5M sucrose, 1.5% (w/v) agar, and supplemented with 5 ng/ml nisin to induce peptide expression. The constructed *L. lactis* strains expressing peptide variants (2-μL overnight culture) were inoculated onto the first-layer GSM17 agar plates, followed by incubation at 30°C for 16–20 h (unless otherwise specified) to promote the growth and peptide production of the host strains before proceeding to the application of the second layer.

For the second-layer preparation, the growth medium varied depending on the specific indicator strain used in the overlay assay. In this study, various indicator strains were employed, including *Lb. sake* ATCC 15521, *E. faecalis, B. subtilis, L. lactis* IL1403 and *Listeria monocytogenes*. The cultivation conditions for these strains were tailored to their specific growth requirements, as described earlier in the “Bacterial strains and growth conditions” section. Typically, each indicator strain was initially cultured in its specific liquid medium to allow for cell enrichment. The resulting enriched cell culture (OD600: 1.0-1.5) was then diluted 1,000-fold into its growth medium supplemented with 0.8% agar at approximately 45°C. Subsequently, the second-layer medium, containing the indicator strain, was poured onto the first-layer GSM17 agar plate where the bacteriocin-producing strain had been grown. Finally, the two-layer testing plate was incubated under the growth conditions described earlier for the specific indicator strain.

### Sequence alignment analysis and structural prediction

To perform sequence alignment analysis, candidate sequences of circular bacteriocins were obtained from the NCBI database and aligned using Clustal Omega (https://www.ebi.ac.uk/Tools/msa/clustalo/). The alignment output was visualized and edited using Jalview (Waterhouse et al., 2009) (version 2.11.2.1). Additionally, the three-dimensional (3-D) peptide structures of circular bacteriocins were modeled in the SWISS-MODEL website (https://swissmodel.expasy.org/interactive), then the PDB formatted files containing the structural information were downloaded. The peptide structure of saposin-C was downloaded from UniProt (https://www.uniprot.org/). These structures were further examined and modified using PyMol (https://pymol.org/2/).

## Results

### Mutational analysis of α-helical replacements between circularin A and enterocin AS-48

**Figure 2:**
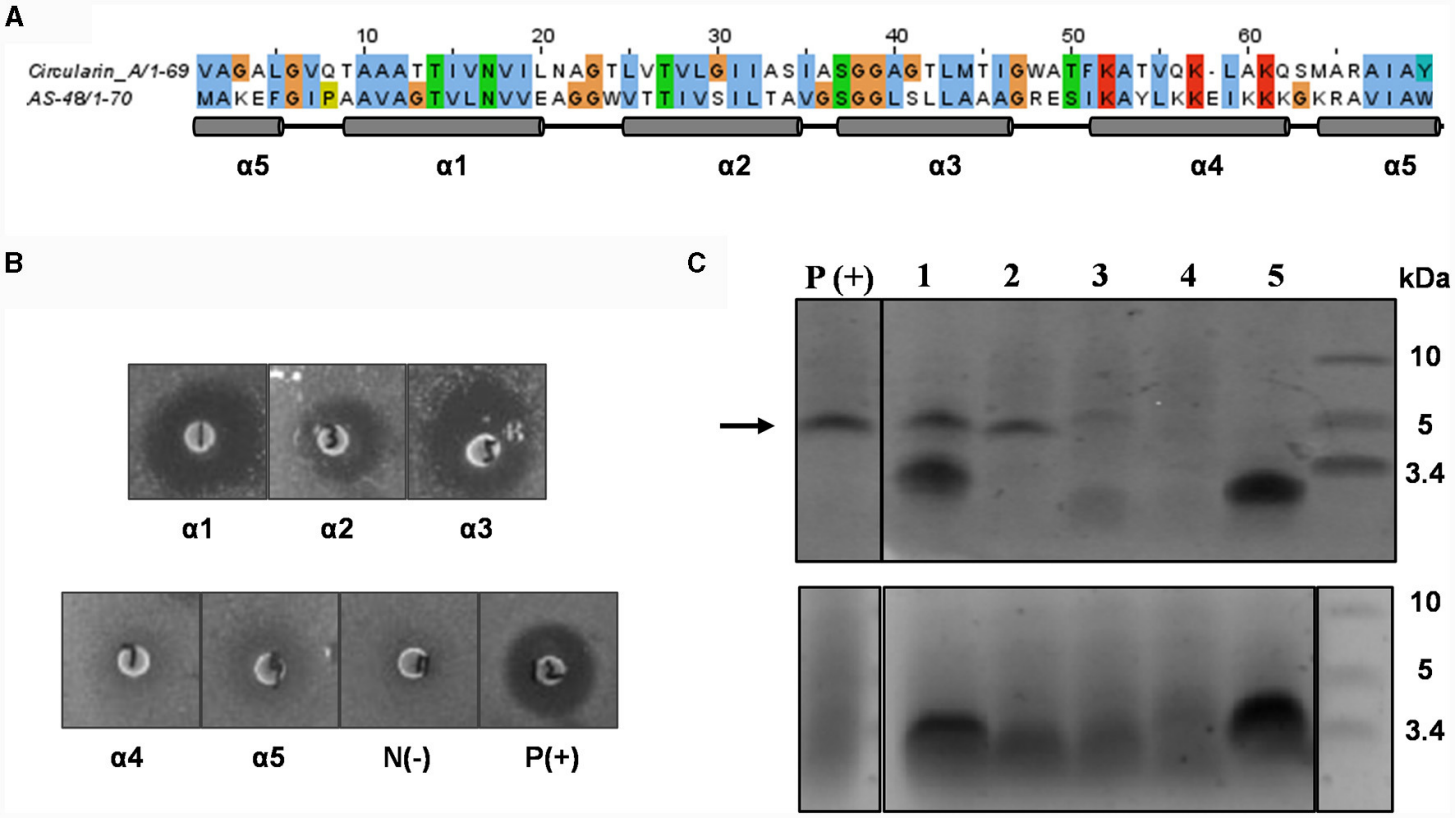
Mutational analysis of α-helix replacement between circularin A and enterocin AS-48. **(A)** Sequence alignment of circularin A and AS-48 with the indication of 5 α-helices based on the AS-48 structure. **(B)** Colony overlay activity assay, including five helical replacements (replacement of α1, α2, α3, α4 and α5, successively) and two control samples [negative control: N(-) and positive control of wild-type circularin A: P(+)]. Indicator strain: *Lactobacillus sake* ATCC 15521. **(C)** SDS-PAGE of purified peptides from constructed α-helix replacement strains: 1, α1 replacement; 2, α2 replacement; 3, α3 replacement; 4, α4 replacement; 5, α5 replacement. P(+) is used as the positive control (wild-type circularin A). Upper panel: target peptides eluted in 80% solvent fractions; lower panel: degraded peptides eluted in 50% solvent fractions. The arrow indicates the size of the mature bacteriocin.

Enterocin AS-48 is the prototype of circular bacteriocins, consisting of 70 amino acids (aa). It belongs to the subgroup I circular bacteriocins and has been extensively investigated and characterized. The elucidated structure of AS-48 demonstrates a saposin-like fold consisting of five α-helices encompassing a hydrophobic core. Circularin A is also a subgroup I circular bacteriocin, consisting of 69 amino acids. Despite the low (~30%) peptide sequence similarity between circularin A and AS-48 (Liu et al., 2022), protein structure modeling suggests that the two bacteriocins share this conserved saposin-like structural motif consisting of five α-helices, and the directionality of their α-helical structures appears to align very well (Figure 1). Based on sequence alignment, the α-helical composition of circularin A was predicted using the elucidated structure of AS-48 as a reference (Figure 2A). Each α-helix of circularin A was replaced with its corresponding α-helix of AS-48 to explore the feasibility of generating hybrid peptides using the biosynthetic machinery of circularin A, and to identify the key structural components responsible for target specificity. Table 1 presents the sequences of the engineered hybrid peptides resulting from the α-helical replacements between circularin A and AS-48.

*Lactobacillus sake* ATCC 15521 was found to be highly sensitive to wild-type circularin A, hence it was chosen as the primary indicator strain for evaluating the antimicrobial activity of all engineered hybrid peptides in this study. For variants with α-helix replacements between circularin A and enterocin AS-48, antimicrobial activity tests revealed that the replacement variants of α1-, α2-, and α3-helix demonstrated antimicrobial activity, whereas the α4 and α5 replacement variants did not exhibit bioactivity (Figure 2B). These findings were supported by the SDS-PAGE analysis (Figure 2C), which showed the presence of the expected band for α1, α2, and α3 replacement variants, but not for α4 and α5 replacement variants. Further analysis with MALDI-TOF showed that the results were generally consistent with the data retrieved from the SDS-PAGE analysis, except for the fact that the target hybrid peptide could not be detected in the α1 replacement variant (Table 2, Supplementary Figure S1). Notably, discrepancies of up to 18 Daltons were observed between the observed and theoretical masses for several peptide variants, including the wild-type circularin A (WT). As reported previously (Liu et al., 2023), we confirmed the simultaneous existence of three mature circularin A forms in the WT strain due to oxidation (Supplementary Figure S2). The differences in observed mass via MALDI-TOF analysis were attributed to the mass averaging of these three forms (WT, WT+16, WT+32). Importantly, in the α1 and α5 replacement variants, we observed partial peptide degradation involving cleavage between M44 and T45 (Figure 2C, Table 2), similar to observations previously reported for some single residue substitutions of CirA (Liu et al., 2023).

**Table 2:** MALDI-TOF analysis of peptide variants following C18 purification, exploring α-helix replacement between circularin A and enterocin AS-48.

**Eluent fractions**	**Mutants**	Sequence^*^	**Theoretical mass**	**Observed mass**	**Mass difference**
80% (full peptide)	WT (circularin A)	*VAGAL*GVQ*TAAATTIVNVIL*NAGT*LVTVLGIIASIASGGAGTLMT* IGWAT*FKATVQKLAKQ*S*MARAIAY*	6,771	6,782	+11
	α1 replacement	VAGALGVQAAVAGTVLNVVENAGTLVTVLGIIASIASGGAGT LMTIGWATFKATVQKLAKQSMARAIAY	6,727	-	-
	α2 replacement	VAGALGVQTAAATTIVNVILNAGTVTTIVSILTAV ASGGAGTL MTIGWATFKATVQKLAKQSMARAIAY	6,789	6,797	+8
	α3 replacement	VAGALGVQTAAATTIVNVILNAGTLVTVLGIIASIASGGLSLLAA IGWATFKATVQKLAKQSMARAIAY	6,765	6,765/6,783	0/+18
50% (degraded 25-aa peptide fragment at the C-terminus)	α1 replacement	TIGWATFKATVQKLAKQSMARAIAY	2,755	2,756	+1
	α5 replacement	TIGWATFKATVQKLAKQSKRA***VIA*****W**	2,803	2,806	+3

^*^The predicted sequences of 5 α-helices in the wild-type circularin A are indicated in italics and underlined. The portions of AS-48 that were introduced by replacing their circularin A counterparts are highlighted in bold and underlined, and the rest of the peptide sequence is from circularin A. It is important to note that due to the circular nature of the peptide, the first and last parts of the peptide form a single α-helix.

The findings from the α1 replacement variant yielded puzzling outcomes, exhibiting successful activity and peptide bands, yet failing in mass detection, necessitating further investigation. In an effort to explore this discrepancy, we prolonged the duration of peptide production in the activity assay before introducing a second layer of the indicator strain *Lb. sake* ATCC 15521. Intriguingly, extended incubation revealed that only the α2 replacement variant exhibited clear activity, whereas the activity of α1 and α3 replacement variants was almost invisible (Supplementary Figure S3). This suggests that although the circularin A biosynthetic machinery can potentially generate the hybrid peptide with the α1 replacement, the resulting peptide might suffer from structural instability. The α4 and α5 replacement variants failed to produce the intended mature peptides, signifying an inability to utilize the circularin A biosynthetic machinery for these structural variants. This resulted in premature degradation of the α4 and α5 replacement peptide variants. Specifically, the α5 replacement variant showed partial degradation, observable as a smaller peptide band in the 50% eluent fractions. In contrast, the α4 replacement variant experienced complete degradation to minute products that couldn't be recognized.

Some AS-48 sensitive strains, such as *Enterococcus faecalis, Bacillus subtilis* and *Listeria monocytogenes*, were also used as indicator strains to test their susceptibility to these engineered hybrid peptides of α-helix replacement between circularin A and enterocin AS-48. However, compared to wild-type circularin A, these α-helix replacement variants did not demonstrate any significant improvement in their effectiveness against the AS-48 sensitive strains (data not shown). Previous studies involving limited proteolysis of AS-48 have reported that the minimal sequence of AS-48 that retained its antimicrobial activity was found to be AS_42,43−10_, which comprises the entire α4 and α5 helices of AS-48 as well as their flanking regions in linear form (Montalbán-López et al., 2008). This may partly explain why α1, α2 and α3 replacement variants did not show similar antimicrobial activity as bacteriocin AS-48. Furthermore, α4 and α5 replacement variants failed to express the engineered hybrid peptides (Figure 2C).

### Mutational analysis of α-helix replacement between circularin A and acidocin B

Acidocin B is a subgroup II circular bacteriocin consisting of 58 amino acids (aa). Its structure has been determined and reveals a folded structure consisting of four α-helices forming an α-helical bundle with a hydrophobic core (Acedo et al., 2015). This type of structure is commonly observed in subgroup II circular bacteriocins. Unlike subgroup I circular bacteriocins that feature a tightly packed saposin-like fold, subgroup II circular bacteriocins contain an α-helical bundle with significantly more surface-exposed hydrophobic patches (Towle and Vederas, 2017). The difference in the arrangement of amphipathic helices between subgroup II and subgroup I circular bacteriocins is responsible for the variation in their structural characteristics. However, both subgroups share a common feature in that the ligation site connecting the N- and C-termini of the bacteriocin occurs within an α-helical structure. Mutational analysis of α-helix replacement between circularin A and acidocin B was performed on the α-helix linking the N- and C- termini of the bacteriocin.

It has been reported that the biosynthetic machinery of circularin A prefers small hydrophobic residues at the N-terminus and aromatic residues at the C-terminus for circularization (Liu et al., 2023). Taking this into account, we designed two mutational variations of α-helix replacement between circularin A and acidocin B. One of the mutants was designed to shift 3 residues from the N-terminus to the C-terminus, resulting in a newly designed peptide starting with a small hydrophobic residue, isoleucine, and ending with an aromatic residue, tryptophan. We also investigated two intermediate mutants that involved replacing either the N-terminus or the C-terminus of circularin A. The detailed sequences of these four mutants (namely M1, M2, M3 and M4) are shown in Table 3.

**Table 3:** Mutational analysis of α-helix replacement between circularin A and acidocin B.

**Variant**	**Abbr**.	Amino acid sequence^*^
Wild-type CirA	WT	MFL- *VAGAL*GVQTAAATTIVNVILNAGTLVTVLGIIASIASGGAGTLMTIGWATFKATVQKLAKQS*MARAIAY*
Mut_C1N1_	M1	MFL-IYWIADQGVQTAAATTIVNVILNAGTLVTVLGIIASIASGGAGTLMTIGWATFKATVQKLAKQSGATAA
Mut_C2N2_	M2	MFL- IADQGVQTAAATTIVNVILNAGTLVTVLGIIASIASGGAGTLMTIGWATFKATVQKLAKQSGATAAIYW
Mut_N2_	M3	MFL- IADQGVQTAAATTIVNVILNAGTLVTVLGIIASIASGGAGTLMTIGWATFKATVQKLAKQSMARAIAY
Mut_C2_	M4	MFL- VAGALGVQTAAATTIVNVILNAGTLVTVLGIIASIASGGAGTLMTIGWATFKATVQKLAKQSGATAAIYW

^*^The presented amino acid sequences are supposed to be the precursor sequences, while the sequence of MFL represents the leader sequence found in the wild-type circularin A. The underlined sequences were subjected to mutational analysis, with bold sequences from acidocin B and the remaining sequences from circularin A. The residues represented by italic sequences form a single α-helix connecting the N- and C-termini of circularin A.

**Figure 3:**
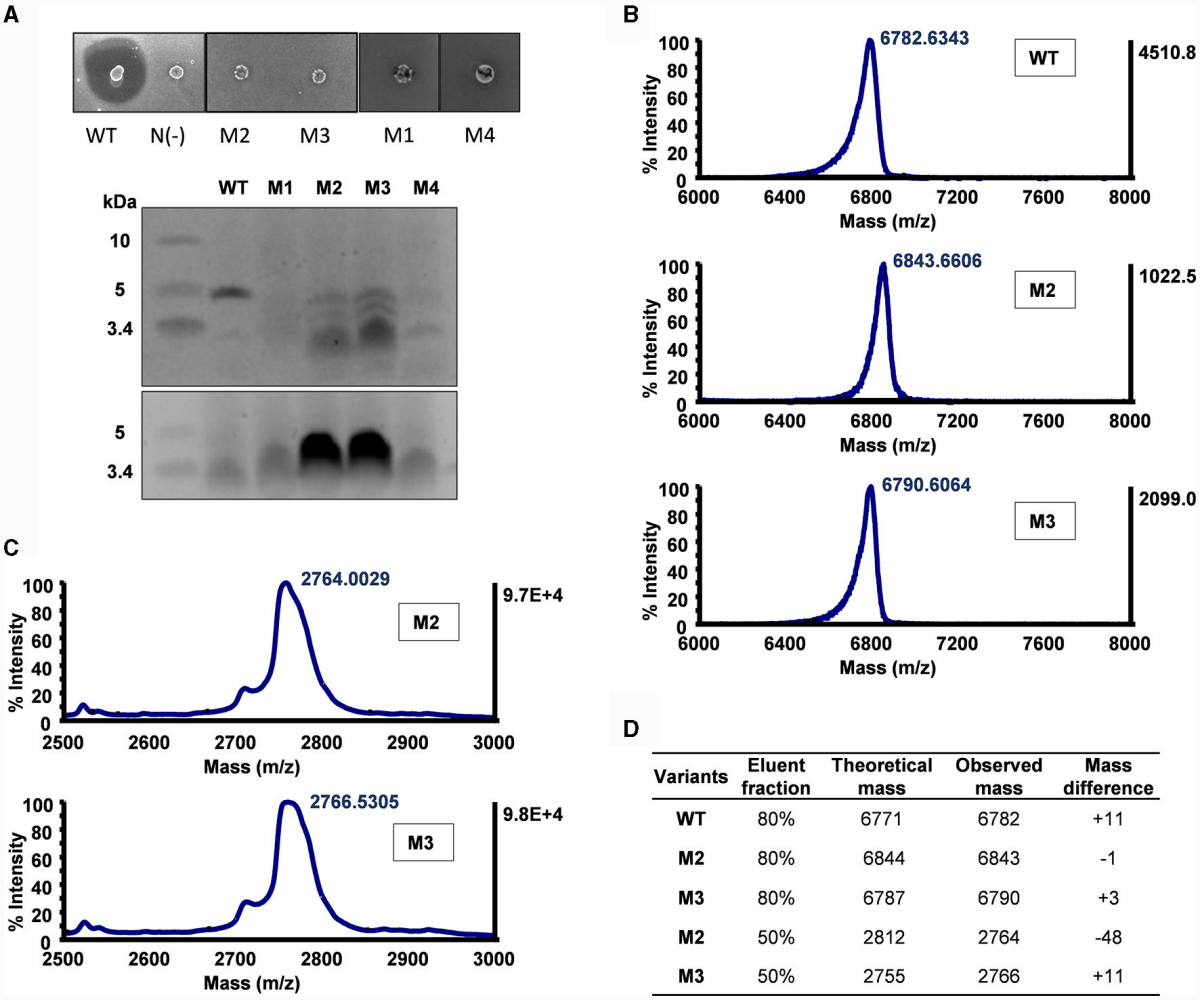
Mutational analysis of α-helix replacement between circularin A and acidocin B. **(A)** Colony overlay activity assay (indicator strain: *Lb. sake* ATCC 15521) and SDS-PAGE analysis (upper panel: target peptides eluted in 80% solvent fractions; lower panel: degraded peptides eluted in 50% solvent fractions). **(B)** MALDI-TOF spectra of the target peptides eluted in 80% solvent fractions. **(C)** MALDI-TOF spectra of the partially degraded peptides eluted in 50% solvent fractions. **(D)** Peptide analysis by comparison between theoretical and observed masses. The mass differences shown in WT and M3 samples are likely due to mixing with their oxidized forms as previously reported (Liu et al., 2023). It would be interesting to further investigate the large difference in M2 (50% elution fraction).

*Lb. sake* ATCC 15521 was used as the indicator strain to assess the antimicrobial activity of the α-helix replacement between circularin A and acidocin B. All of these mutants appeared to have completely lost antimicrobial activity, with the exception of M3 which still displayed a minimal inhibition (Figure 3A). These mutants also exhibited indications of mild growth stress, as evidenced by their altered colony morphology compared to the wild-type strain. According to the SDS-PAGE analysis, only M2 and M3 mutants exhibited peptide bands of similar size to the wild-type peptide, albeit with significantly lower intensity, while the other two mutants did not show any such bands. The difference in the sequences of M1 and M2 involves the relocation of three residues from the N-terminus to the C-terminus in the latter mutant. This result aligns with the previously reported importance of aromatic residues in the last position for bacteriocin processing (e.g., circularization) by the circularin A biosynthetic machinery (Liu et al., 2023).

The M3 and M4 mutants are intermediate mutants of M2, involving substitution of the N-terminus and the C-terminus of circularin A, respectively. The M3 mutant exhibited similar results as M2, revealing the production of the designed hybrid peptide, as well as the partially degraded peptide (Figure 3). This is consistent with earlier studies reporting that substitution of N-terminal residues could reduce the modification efficiency and result in partial degradation (Liu et al., 2023). The M4 mutant failed to produce the intended hybrid peptide, which may indicate that the replaced C-terminal segment and the native N-terminal portion of circularin A may not be compatible within a single integrated α-helix.

### Mutagenesis studies of introducing potential disulfide bonds in circularin A

**Figure 4:**
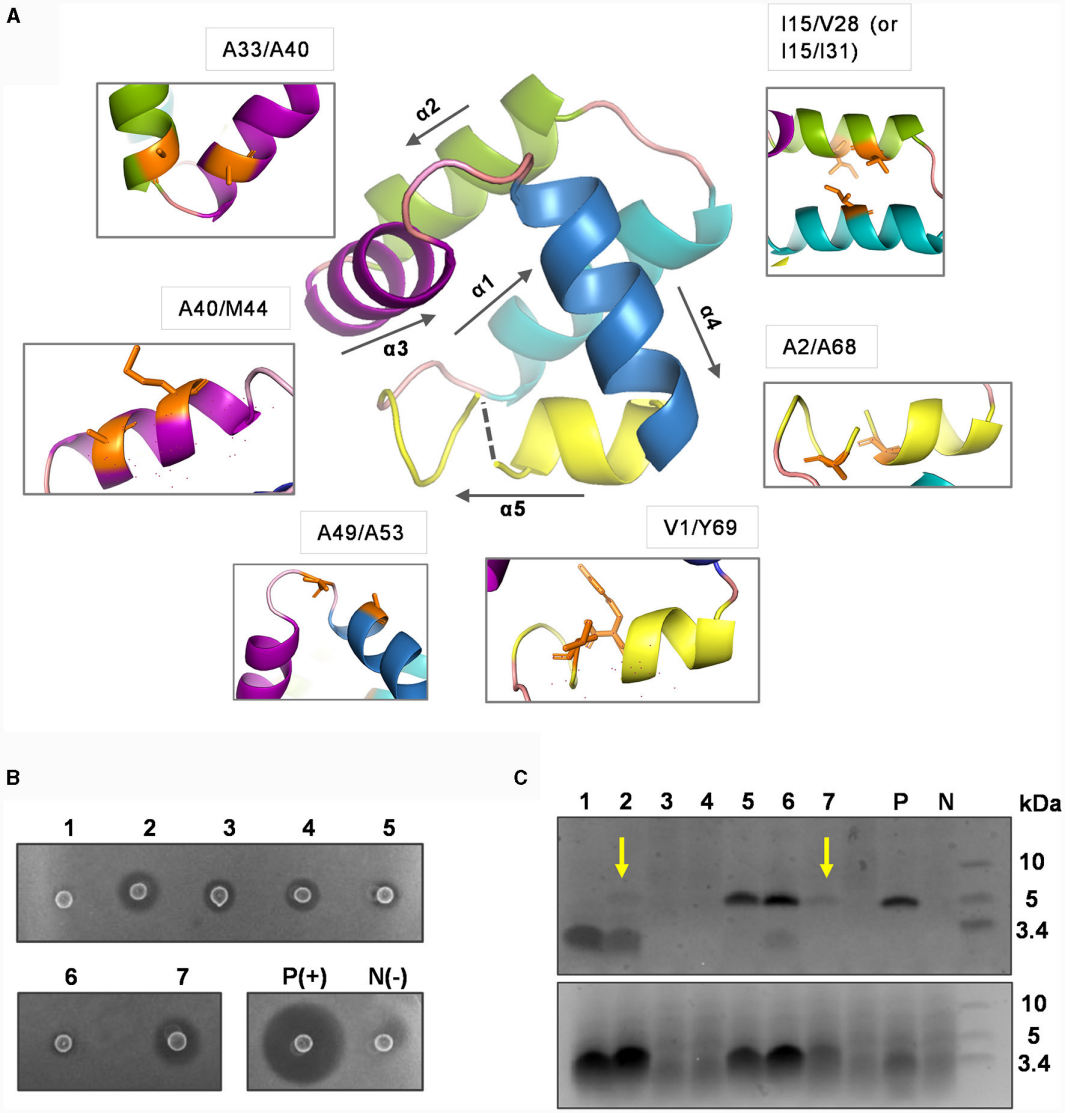
Mutational analysis of introducing potential disulfide bonds in circularin A. **(A)** The residues subjected to cystine substitutions in circularin A (indicated by orange sticks). The peptide structure of circularin A was first modeled in the SWISS-MODEL website, and then further examined and edited using PyMol. **(B)** Colony overlay activity assay (indicator strain: *Lb. sake* ATCC 15521): 1, V1C/Y69C; 2, A2C/A68C; 3, I15C/V28C; 4, I15C/I31C; 5, A33C/A40C; 6, A40C/M44C; 7, A49C/A53C; P(+), positive control (wild-type circularin A) and N(-), negative control. **(C)** SDS-PAGE analysis (upper panel: target peptides eluted in 80% solvent fractions; lower panel: degraded peptides eluted in 50% solvent fractions). The samples are numbered in the same order as in the **(B)**. The yellow arrows indicate the faint target bands observed for samples No. 2 (A2C/A68C) and No. 7 (A49C/A53C). P and N represent the positive and negative controls, respectively.

Circular bacteriocins are characterized by head-to-tail ligation. They have a three-dimensional configuration that features either a saposin-like fold or a closely related α-helical bundle (Towle and Vederas, 2017). Unlike other types of circular peptides (e.g., plant-originated cyclotides) and saposins that rely on disulfide bonds to maintain their three-dimensional structure, circular bacteriocins generally lack cysteine residues. Instead, hydrophobic side chain interactions are thought to play a role in the stabilization of circular bacteriocins upon peptide circularization (Martin-Visscher et al., 2009; Gor et al., 2020). To examine the impact of introducing potential disulfide bonds into circularin A, which restrict dynamic movements of the α-helices, on its biosynthesis and bioactivity, a total of seven variants were generated (Table 4). These variants included potential disulfide bonds for intra- or inter-α-helix linkage (variants No. 2-6), linkage between loop and α-helix (variant No. 7), as well as cysteine substitutions at the original circularization site (variant No. 1) (Table 4, Figure 4A).

**Table 4:** Mutational analysis of introducing potential disulfide bonds in circularin A.

**No**.	**Mutants**	**Description (potential linkage)**
1	V1C/Y69C	Circularization site; α5 intralinkage
2	A2C/A68C	α5 intralinkage
3	I15C/V28C	α1 and α2
4	I15C/I31C	α1 and α2
5	A33C/A40C	α2 and α3
6	A40C/M44C	α3 intralinkage
7	A49C/A53C	Loop and α4

The activity tests, combined with SDS-PAGE analysis, revealed that these variants had dramatic impacts on bacteriocin biosynthesis and/or bioactivity (Figures 4B, C). Specifically, the majority of these variants (except variants No. 5 and No. 6) had simultaneous effects between activity and production yields: variants No. 2 and No.7 (A2C/A68C and A49C/A53C) exhibited faint target bands and thus had higher activity levels compared to variants No. 3, and No. 4 (I15C/V28C and I15C/I31C), which did not show target bands. Variant No. 1 (V1C/Y69C), where cysteine substitutions were made for residues at the ligation site, showed no detectable activity, indicating that circularization may have failed. Although variants No. 5 and No. 6 (A33C/A40C and A40C/M44C) demonstrated peptide yields comparable to wild-type circularin A, their activity was very low. To determine whether the decreased activity observed in variants No. 5 and No. 6 was a result of structural constraints due to disulfide bond formation, their purified peptide variants were first subjected to treatment with the reducing agent tris(2-carboxyethyl) phosphine (TCEP) and then evaluated for antimicrobial activity. When compared to samples without TCEP treatment, the No. 5 peptide variant (A33C/A40C) exhibited enhanced activity after being treated with TCEP, whereas no significant difference was observed for the No. 6 peptide variant (A40C/M44C) (Supplementary Figure S4). This likely indicated that the disulfide bond was successfully formed in the variant No. 5 (A33C/A40C), linking the α2 and α3 helices of circularin A, which might have restricted the flexibility of the bacteriocin and resulted in reduced antimicrobial activity. Notably, enterocin AS-48 has been reported to exist in dimeric forms in solutions, namely the water-soluble DF-I and the membrane-bound DF-II (Grande Burgos et al., 2014). The dynamic flexibility of AS-48, allowing rearrangement and transition from DF-I to DF-II at the membrane surface, is believed to be crucial for its ability to insert into bacterial membranes and form pores (Grande Burgos et al., 2014; Cebrián et al., 2023). In the case of variant No. 6 (A40C/M44C), the loss of activity was more likely associated with the introduced mutations rather than structural constraint caused by disulfide bond formation.

## Discussion

This study aimed to explore the bacteriocin engineering potential by utilizing the biosynthetic machinery of a circular bacteriocin, circularin A. Throughout this investigation, we investigated various aspects encompassing α-helix replacements, disulfide bond introductions, and the subsequent impact on the biosynthesis, bioactivity, and structural stability of circularin A and related circular bacteriocins. First, we explored the feasibility of α-helix replacements between subclass I circular bacteriocins AS-48 and circularin A. The results illustrated that substitutions of the internal helices α1, α2 and α3 proved viable for bacteriocin processing and maturation utilizing circularin A biosynthetic proteins, whereas the substitution of the N- and C- connecting helix α5 failed to produce the intended engineered peptide. Through careful experimental designs, N- and C- connecting helix replacements between circularin A and subclass II circular bacteriocin, acidocin B, were successfully produced. This indicates a promising potential for engineering circular bacteriocins, while also highlighting the inherent challenges involved in modifying these bacteriocins through peptide engineering strategies. Contrary to the expectations based on the significance of disulfide bonds in many biological peptides, the introduction of disulfide bonds in circularin A yielded adverse effects, significantly reducing bacteriocin biosynthesis and/or bioactivity. This highlights the vital role of dynamic flexibility within the helices of circularin A.

A notable observation throughout this study is the prevalence of partial degradation. This observation aligns with prior studies that highlight how substituting N-terminal residues tends to diminish modification efficiency and result in partial degradation. It is apparent that mutations induce varying levels of modification efficiency, often reducing efficiency, thereby prolonging the time the peptide remains in its linear form within the cell. Consequently, this extended duration elevates susceptibility to degradation by host peptidases. Depending on the structural alterations dictated by primary sequence changes, mutations in circularin A could result in degradations including either partial or complete degradation of the variant peptide. Minor alterations frequently lead to partial degradation, exemplified by the observed cleavage between the M44-T45 linkage of the circularin A peptide, yielding a 25-amino-acid C-terminal peptide fragment. Conversely, significant changes often result in the complete degradation of the precursor peptide. Future optimization endeavors should consider these factors to enhance the modification rate and achieve increased peptide yield.

In conclusion, this study presents an unprecedented exploration into the realm of circular bacteriocin engineering, specifically focusing on the bacteriocin circularin A. We successfully achieved the production of various α-helix replacements between circularin A and other circular bacteriocins (e.g., enterocin AS-48 and acidocin B). Although some substitutions appear not to be very stable, our results demonstrate various possibilities for peptide engineering to alter properties of circular bacteriocins. While disulfide bonds play important roles in other biological peptides, the introduction of disulfide bonds in circularin A greatly reduced bacteriocin biosynthesis and/or bioactivity. This highlights the importance of dynamic flexibility of the respective helices in circularin A. Overall, these findings represent a promising starting point for peptide engineering of circular bacteriocins and provide valuable insights into utilizing circular bacteriocins as scaffolds for introducing certain properties. Moreover, this study sheds light on the engineering potential of circular bacteriocins as antimicrobial agents and the structural and functional limitations within the peptide sequence. Further research is necessary to explore the engineering potential of circularin A and other circular bacteriocins in this context. By understanding these limitations, researchers can better explore and optimize the design of circular bacteriocins for enhanced antimicrobial activity and/or target specificity.

## Data availability statement

The original contributions presented in the study are included in the article/Supplementary material, further inquiries can be directed to the corresponding author.

## Author contributions

FL: Conceptualization, Data curation, Formal analysis, Funding acquisition, Investigation, Methodology, Software, Validation, Visualization, Writing – original draft, Writing – review & editing. AJvH: Conceptualization, Writing – review & editing. OPK: Conceptualization, Project administration, Supervision, Writing – review & editing, Resources, Funding acquisition.
