# Supplementary material for: Engineering circular bacteriocins: structural and functional effects of α-helix exchanges and disulfide introductions in circularin A
Source: Front Microbiol. 2024 Feb 15;15:1337647. doi: 10.3389/fmicb.2024.1337647 (PMC10905743; doi:10.3389/fmicb.2024.1337647)
Supplement: Supplementary file 1 [file Data_Sheet_1.PDF]

Supplementary Material

Engineering circular bacteriocins: structural and functional effects of α-helix exchanges and disulfide introductions in circularin A

Fangfang Liu[a], Auke J. van Heel[a,b] and Oscar P. Kuipers[a,b,*]

[a]Department of Molecular Genetics, Groningen Biomolecular Sciences and Biotechnology Institute, University of Groningen, 9747 AG Groningen, the Netherlands

[b]Omnicin Therapeutics, Groningen, the Netherlands

*** Correspondence:**
Oscar P. Kuipers
o.p.kuipers@rug.nl

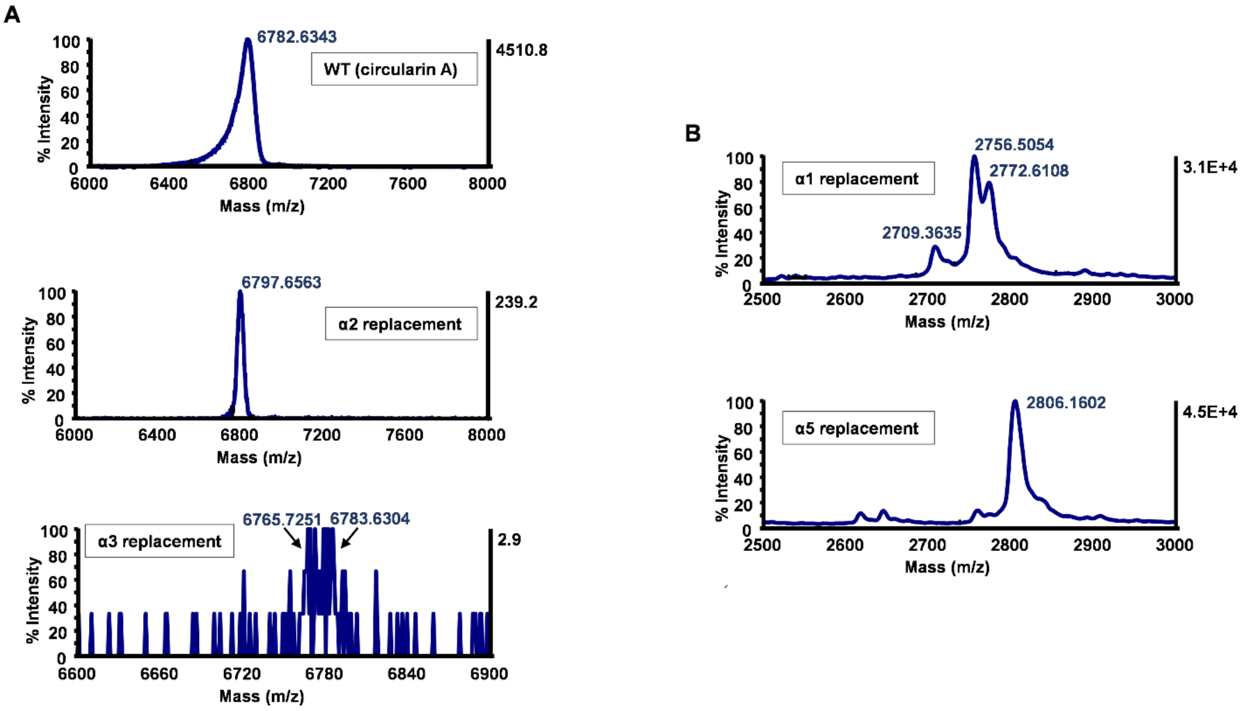

Figure S1. Peptide analysis via mass spectrometry conducted on mutants featuring α-helix replacement between circularin A and AS-48 following C18 purification. (A) MALDI-TOF spectra of purified peptides from the 80% solvent-eluted fractions for detecting the full peptides (only α2 replacement mutant and α3 replacement mutant showed masses corresponding to the intended target peptides). (B) MALDI-TOF spectra of purified peptides from 50% solvent-eluted fractions for detecting the degraded peptides (α1 replacement mutant and α5 replacement mutant showed masses corresponding to partially degraded peptides).

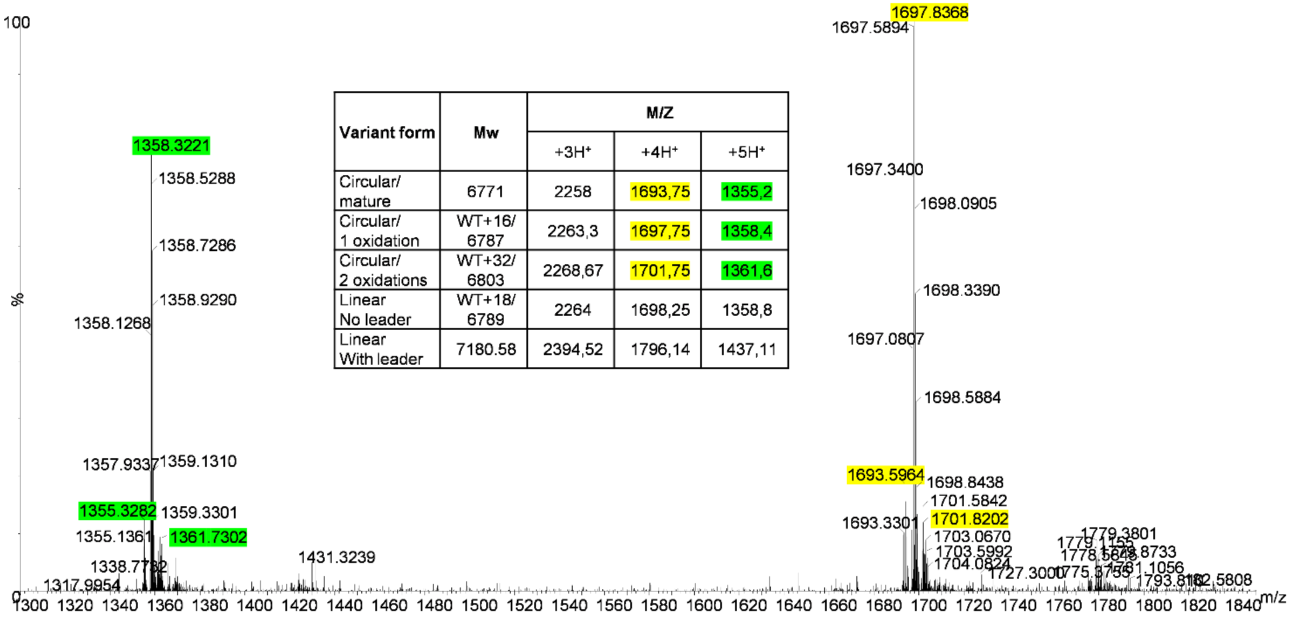

Variant form	Mw	M/Z		
		+3H+	+4H+	+5H+
Circular/ mature	6771	2258	1693,75	1355,2
Circular/ 1 oxidation	WT+16/ 6787	2263,3	1697,75	1358,4
Circular/ 2 oxidations	WT+32/ 6803	2268,67	1701,75	1361,6
Linear No leader	WT+18/ 6789	2264	1698,25	1358,8
Linear With leader	7180.58	2394,52	1796,14	1437,11

Figure S2. Masses detected via LC-MS analysis of the wild-type circularin A revealed three fully modified forms: the circular mature bacteriocin (WT), WT with one oxidation, and WT with two oxidations.

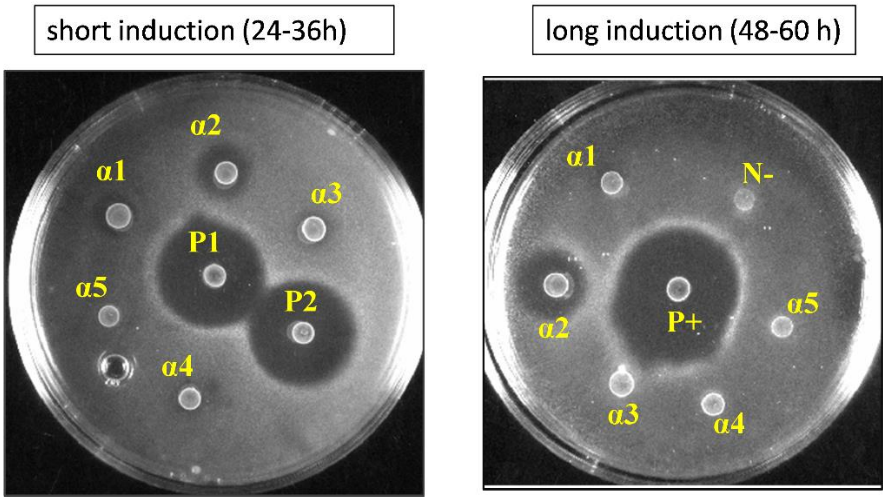

Figure S3. The colony overlay activity assay, including five helical replacements (replacement of α1, α2, α3, α4 and α5, respectively) and two control samples (negative control: N(-) and positive control of wild-type circularin A: P(+)). Indicator strain: *Lactobacillus sake* ATCC 15521.

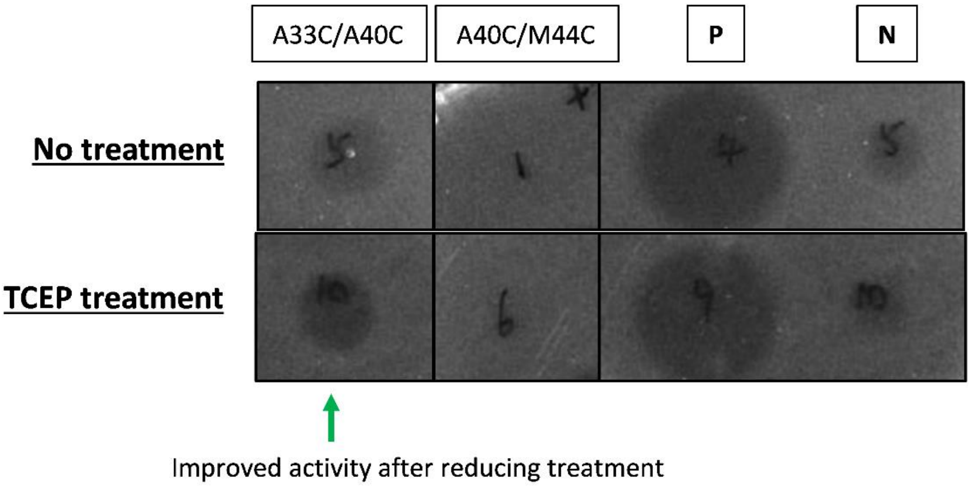

Figure S4. Activity comparison of peptide variants before and after TCEP treatment. P and N represent the positive control (indicating the presence of wild-type circularin A) and negative control (indicating the absence of wild-type circularin A), respectively. The samples A33C/A40C and A40C/M44C represent corresponding mutants of circularin A.

Table S1. Oligonucleotides used in this study

**Constructs**		**Primer**	**Sequence (5'→3')**
1) α-helix replacement helix replacement			
CirA/ AS-48	α1-helix	P01	GCTGGTACTGTTCTTAACGTTGTTGAAAATGCAGGTACATTAGTTACTGTTTT
		P02	CAACGTTAAGAACAGTACCAGCAACAGCAGCTTGCACGCCTAGTGCTCC
	α2-helix	P03	CTATCGTTTCAATCCTTACTGCTGTTGCAAGTGGTGGTGCAGG
		P04	CAGTAAGGATTGAAACGATAGTAGTAACTGTACCTGCATTAAGAATAACATT
	α3-helix	P05	GGTGGTCTTTCACTTCTTGCTGCTATTGGATGGGCAACATTCAAAG
		P06	AGCAAGAAGTGAAAGACCACCTGATGCAATTGAAGCTATAATACCTAAAAC
	α4-helix	P07	CTTACCTTAAAAAAGAAATCAAAAAAAAAAGTATGGCAAGAGCTATAGCTT
		P08	GATTTCTTTTTTAAGGTAAGCTTTGATTGTTGCCCATCCAATAGTC
	α5-helix	P09	ATGGCTAAAGAATTCGGCGTGCAAACAGCTGC
		P10	CTTACCAAGCGATAACAGCACGTTTACTTTGCTTAGCTAATTTTTGAACTGTT
		P11	AAACGTGCTGTTATCGCTTGGTAAGCTTTCTTTGAACCAAAATTAGAAAACC
		P12	GCACGCCGAATTCTTTAGCCATTAAAAACATGGTGAGTGCCTCC
CirA/ AciB	M1	P13	GTTCAAAAATTAGCTAAGCAAAGTGGTGCTACTGCTGCTTAAGCTTTCTTTGA ACCAAAATTAGAAAACC
		P14	TAGCTGCAGCTGTTTGCACGCCGAATTGATCAGCGATCCAGTAGATTAAAAA CATGGTGAGTGCCTCC
	M2	P15	GTTCAAAAATTAGCTAAGCAAAGTGGTGCTACTGCTGCTATCTACTGGTAAG CTTTCTTTGAACCAAAATTAGAAAACC
		P16	TAGCTGCAGCTGTTTGCACGCCGAATTGATCAGCGATTAAAAACATGGTGAG
2) cysteine substitutions (introducing potential disulfide bonds)			
V1C/Y69C		P24	ATGTTTTTATGTGCAGGAGCACTAGGCGTG
		P25	CCTAGTGCTCCTGCACATAAAAACATGGTGAG
		P26	AGAGCTATAGCTTGTTAAGCTTTCTTTGAACCAAAA
		P27	GTTCAAAGAAAGCTTAACAAGCTATAGCTCTTGCCAT
A2C/A68C		P28	ATGTTTTTAGTTTGTGGAGCACTAGGCGTGCAAAC
		P29	ACGCCTAGTGCTCCACAAACTAAAAACATGGTG
		P30	TATGGCAAGAGCTATATGTTACTAAGCTTTCTTTGAACCAA
		P31	GTTCAAAGAAAGCTTAGTAACATATAGCTCTTGCCATACTT
I15C/V28C		P32	CTTAATGCAGGTACATTAGTTACTTGTTTAGGTATTATAGCTTC
		P33	AACTAATGTACCTGCATTAAGAATAACATTAACACATGTAGTAGCTG
I15C/I31C		P34	CTTAATGCAGGTACATTAGTTACTGTTTTAGGTTGTATAGCTTCAA
		P33	AACTAATGTACCTGCATTAAGAATAACATTAACACATGTAGTAGCTG
A33C/A40C		P35	TGTTCAATTGCAAGTGGTGGTTGTGGTACATTAATGACTATTGG
		P36	ACAACCACCACTTGCAATTGAACATATAATACCTAAAACAGTAAC
A40C/M44C		P37	GGTTGTGGTACATTATGTACTATTGGATGGGCAACATTCAAAGC
		P38	GTACATAATGTACCACAACCACCACTTGCAATTGAAGC
A49C/A53C		P39	GGTGTACATTCAAATGTACAGTTCAAAAATTAGCTAAGCAAAGTATGGC
		P40	CTGTACATTTGAATGTACACCATCCAATAGTCATTAATGTACCTGCAC
